# Apples and oranges: avoiding different priors in Bayesian DNA sequence analysis

**DOI:** 10.1186/1471-2105-11-149

**Published:** 2010-03-22

**Authors:** Jens Keilwagen, Jan Grau, Stefan Posch, Ivo Grosse

**Affiliations:** 1 Molecular Genetics, Leibniz Institute of Plant Genetics and Crop Plant Research (IPK), Gatersleben, Germany; 2 Institute of Computer Science, Martin Luther University Halle-Wittenberg, Halle/Saale, Germany

## Abstract

**Background:**

One of the challenges of bioinformatics remains the recognition of short signal sequences in genomic DNA such as donor or acceptor splice sites, splicing enhancers or silencers, translation initiation sites, transcription start sites, transcription factor binding sites, nucleosome binding sites, miRNA binding sites, or insulator binding sites. During the last decade, a wealth of algorithms for the recognition of such DNA sequences has been developed and compared with the goal of improving their performance and to deepen our understanding of the underlying cellular processes. Most of these algorithms are based on statistical models belonging to the family of Markov random fields such as position weight matrix models, weight array matrix models, Markov models of higher order, or moral Bayesian networks. While in many comparative studies different learning principles or different statistical models have been compared, the influence of choosing different prior distributions for the model parameters when using different learning principles has been overlooked, and possibly lead to questionable conclusions.

**Results:**

With the goal of allowing direct comparisons of different learning principles for models from the family of Markov random fields based on the *same a-priori information*, we derive a generalization of the commonly-used product-Dirichlet prior. We find that the derived prior behaves like a Gaussian prior close to the maximum and like a Laplace prior in the far tails. In two case studies, we illustrate the utility of the derived prior for a direct comparison of different learning principles with different models for the recognition of binding sites of the transcription factor Sp1 and human donor splice sites.

**Conclusions:**

We find that comparisons of different learning principles using the same a-priori information can lead to conclusions different from those of previous studies in which the effect resulting from different priors has been neglected. We implement the derived prior is implemented in the open-source library Jstacs to enable an easy application to comparative studies of different learning principles in the field of sequence analysis.

## Background

The computational recognition of short signal sequences in genomic DNA is one of the prevalent tasks in bioinformatics. It includes e.g. the recognition of transcription factor binding sites (TFBSs) [1,2], donor or acceptor splice sites [3-5], nucleosome binding sites [6,7], or binding sites of insulators like CTCF [8]. Many different algorithms have been developed for the recognition of such DNA binding sites, with specific strengths and weaknesses, but none of them is perfect. Hence, great efforts have been made over the last decade to evaluate and compare the performance of different algorithms [2,3,9-13]. The results of such comparative studies are often influential to the direction of future research, because they lead to new and superior approaches by combining the advantages of existing algorithms and because they provide a deeper understanding of the mechanisms of protein-DNA interaction. The approaches compared typically differ by (i) the statistical model employed at the heart of these algorithms, (ii) the learning principle chosen for estimating the model parameters, and (iii) the prior used for the parameters of the model, and it is non-trivial to keep the influences of these different contributions apart. The first two aspects focus on developing improved statistical models or learning principles, while the choice of the prior is often arbitrary or determined by conjugacy. However, the choice of the prior may have a decisive effect on the recognition performance [14,15]. The goal of this paper is to derive a common prior for Markov random fields (MRFs) and mixtures of MRFs, which are at the heart of many existing algorithms for binding site recognition, allowing an unbiased comparison of different learning principles for models from this model family.

Many computer algorithms available today use statistical models for representing the distribution of sequences, and many of these statistical models are special cases of MRFs [16,17]. These models range from simple models like the position weight matrix (PWM) model [1,18,19], the weight array matrix (WAM) model [4,6,20], or Markov models of higher order [21,22] to more complex models like moral Bayesian networks [2,12,23] or general MRFs [5,24,25]. Hence, we restrict our attention to statistical models from the family of MRFs in this paper.

One of the first learning principles used in bioinformatics is the maximum likelihood (ML) principle. However, for many applications, the sequence data available for learning statistical models is very limited. This is especially true for the recognition of TFBSs, where typical data sets contain sometimes as few as 20 and seldom more than 300 sequences. For this reason, the ML principle often leads to suboptimal classification performance e.g. due to zero-occurrences of some nucleotides or oligonucleotides in the training data sets. The maximum a-posteriori (MAP) principle, which applies a prior to the parameters of the models, establishes a theoretical foundation to alleviate this problem and at the same time allows for the inclusion of prior knowledge aside from the training data.

Recently, the application of discriminative principles instead of generative ones has been shown to be promising in the field of bioinformatics [9,21,22,24,26]. Generative learning principles aim at an accurate representation of the distribution of the training data, whereas discriminative learning principles aim at an accurate classification of the training data. The discriminative analogue to the ML principle is the maximum conditional likelihood (MCL) principle, which has been widely used in the machine learning community [27-31]. However, the effects of limited data may be even more severe when using the MCL principle compared to generative learning principles [11]. To overcome this problem, the maximum supervised posterior (MSP) principle [32,33] has been proposed as discriminative analogue to the MAP principle.

Many different priors have been used in the past, and their choice seems arbitrary or motivated by technical aspects. Product-Gaussian and product-Laplace priors are widely used for generatively trained MRFs [16] and discriminatively trained MRFs also called conditional random fields [17,34]. For the generative MAP learning of Markov models and Bayesian networks, the most prevalent prior is the product-Dirichlet prior, whereas for the discriminative MSP learning, either a product-Gaussian or product-Laplace prior is typically employed [26]. Hence, when comparing generatively and discriminatively trained Markov models, Bayesian networks, and MRFs, in many occasions apples are compared to oranges by using different priors.

The comparison of generative and discriminative learning principles is the topic of several recent studies. Ng & Jordan [11] compare generatively and discriminatively trained PWM models. To be specific, they compare the Bayesian MAP principle with the non-Bayesian MCL principle. Pernkopf & Bilmes [30] compare the ML principle to the MCL principle for estimating the parameters of Bayesian networks, while the structures of the networks are estimated by generative as well as discriminative measures. Greiner et al. [29] compare the ML principle with a variant of the MCL principle that prevents over-fitting, and they apply these approaches to Bayesian networks. Grau et al. [26] compare the MAP principle for Markov models using a product-Dirichlet prior to the MSP principle using product-Gaussian and product-Laplace priors.

All of these studies use *different *priors when comparing different learning principles, rendering the conclusions regarding the superiority of one learning principle over the other questionable, because the *differing *influences of these priors are neglected. In fact, we are not aware of any study that uses the same a-priori information when comparing generative to discriminative learning principles.

Motivated by this lack of consistency, we aim at establishing a prior that

i) can be used for the generative (MAP) and the discriminative (MSP) principles,

ii) is conjugate to the likelihood of MRFs, which include moral Bayesian networks,

iii) contains the widely-used product-Dirichlet prior as special case when the structure of the MRF is equivalent to that of a moral Bayesian network including all of its special cases such as PWM models, WAM models, Markov models of higher order, or Bayesian trees.

In section *Methods*, we present the derivation of such a prior, which is the main result of this paper. With such a prior at hand, it becomes possible to accomplish an unbiased comparison of generative and discriminative learning principles applied to the same model using the same prior. In addition, this prior allows a comparison of different generatively trained models for binding site recognition that are special cases of MRFs including PWM models, WAM models, Markov models of higher order, Bayesian trees, or moral Bayesian networks as well as a comparison of different discriminatively trained models that are special cases of MRFs using the BDeu prior [35]. In section *Results and Discussion*, we illustrate the applicability of the derived prior using two typical data sets of TFBSs and donor splice sites.

## Methods

We denote by *x*= (*x*_1_, ..., *x*_*L*_) a sequence of length *L *over an alphabet Σ = {1, 2, ..., *S*} with *x*_ℓ _∈ Σ, where *S *= 4 in case of DNA and RNA sequences, and *S *= 20 in case of protein sequences. We denote by *c *∈  = {1, 2, ..., *C*} the class of a sequence. In this paper, we consider two-class problems, i.e., *C *= 2, and we denote the first class containing biological binding sites by *foreground*, and the second class containing decoy DNA sequences by *background*. For each sequence *x*_*n *_in the training data set, we know its correct class label *c*_*n *_∈ . We denote the data set of all sequences by  = (*x*_1_, ..., *x*_*N*_) and we denote the vector of the corresponding class labels by *c*= (*c*_1_, ..., *c*_*N*_).

In this paper, we consider two Bayesian learning principles, namely the generative maximum a-posteriori (MAP) principle and the discriminative maximum supervised posterior (MSP) principle. The goal of both learning principles is to estimate the optimal parameters of some statistical model with respect to the posterior or supervised posterior, respectively.

Using the MAP principle, the parameters ϑ are optimized with respect to the posterior, which is proportional to the product of a parameter prior *h *(ϑ|*α*) given hyper-parameters *α* and the likelihood *p*(, *c*|ϑ) of the data set  and the class labels *c*given parameters ϑ:(1a)

Under the assumption of independent and identically distributed (i.i.d.) data, we obtain(1b)

Using the assumption of i.i.d. sequences and the assumption of independence of the parameters of the classes, generative learning principles, as for instance the MAP principle, can be simplified to class-specific generative learning principles that allow inferring the parameters of the foreground and background class separately. For several simple models like Markov models, generative learning principles amount to computing smoothed relative frequencies of nucleotides and oligonucleotides [18-20].

For the MSP principle, the parameters ϑare optimized with respect to the supervised posterior, which is defined as the product of a parameter prior *h*(ϑ|*α*) given hyper-parameters *α*and the conditional likelihood *p *(*c*, *λ*) of the class labels *c*given the data set  and parameters ϑ:(2a)

We again assume i.i.d. data and express the class posteriors *p* (*c*_*n*_|*x*_*n*_, *λ*) in terms of likelihoods *p* (*c*,*x*_*n*_|*λ*), yielding(2b)

While the generative ML and MAP principles often lead to analytic solutions for simple models such as Markov models, we must use numerical optimization procedures [36] for the discriminative MCL and MSP principles.

In practical applications, the parameterization ϑof the models and the priors *h*(ϑ|*α*) differ between the MAP and the MSP principle, since both learning principles evolved from different theoretical backgrounds. With the goal of resolving these differences, we present a common parameterization for the likelihood of all models from the class of MRFs, which can be used for the MAP and the MSP principle, and we derive a prior for this parameterization that is equivalent to the well-known product-Dirichlet prior in the remainder of this section.

### Foundations of moral Bayesian networks

Graphical models, which combine probability theory and graph theory, are statistical models in which random variables are represented by nodes of a graph and in which the dependency structure of the joint probability distribution is represented by edges [37]. Graphical models can be categorized into *directed *acyclic graphical models called Bayesian networks and *undirected *graphical models called MRFs with a non-empty intersection called moral Bayesian networks [38]. For deriving the desired prior, we start with moral Bayesian networks in this subsection, where we give an introduction to moral Bayesian networks, and in the second subsection we present the MRF parameterization for these models. In the third subsection, we present the widely-used product-Dirichlet prior for moral Bayesian networks, and transform this prior to the MRF parameterization. Finally, we extend the resulting prior for moral Bayesian networks to the case of general MRFs in the last subsection.

Graphical models are represented by graphs consisting of nodes and edges. The nodes in the graph represent random variables *X*_ℓ_ having realizations denoted by *x*_ℓ_. In case of directed graphical models, the edges are directed from the *parent *nodes to their *children*. We denote by Pa(ℓ) the vector of parents of node ℓ representing random variable *X*_ℓ_, and we denote by pa(ℓ, *x*) the realizations of the parents Pa(ℓ) in sequence *x*. Edges between nodes represent potential statistical dependencies between the random variables, while missing edges between nodes represent conditional independencies of the associated random variables given their parents. Specifically, if there is no edge from *i *to *j*, then *X*_*i*_ and *X*_*j *_are conditionally independent given Pa(*i*) and Pa(*j*), the parents of node *i* and *j*. For Bayesian networks the underlying graph structure is a directed acyclic graph (DAG). In this paper, we consider models with a given graph structure, such that all parents of each node are pre-determined. To simplify notation in the following derivation, we assume the same graph structure for the models of all classes. The extensions to models with different graph structures and to position-dependent alphabets is straightforward.

A Bayesian network is called a *moral* Bayesian network iff its DAG is moral. A DAG is called moral iff, for each node ℓ, each pair (*p*_1_, *p*_2_), *p*_1_ ≠ *p*_2_, of its parents is connected by an edge [38]. The family of moral Bayesian networks contains popular models such as PWM models, WAM models, Markov models of higher order, and Bayesian trees. When considering the parents Pa(ℓ) of a node ℓ in a moral Bayesian network, we can order the nodes in Pa(ℓ) uniquely according to the topological ordering within the set Pa(ℓ).

With these prerequisites, we present the likelihood of a moral Bayesian network in a parameterization that is often used for the MAP principle. In the following, we denote these parameters by *θ*compared to ϑ in equation (1a). The likelihood *p*_*θ*_(*x*, *c*|*θ*) of a moral Bayesian network with parameters *θ*is defined by(3)

where *θ*_*c *_denotes the probability of class *c*, and  denotes the probability of observing *x*_ℓ _at *X*_ℓ _in class *c *given the observations pa(ℓ, *x*) at the random variables represented by the nodes Pa(ℓ) [39]. The following constraints together with the non-negativity of the *θ*-parameters ensure that subsets of the components of *θ *remain on simplices:

with *c *∈ , ℓ ∈ [1, *L*], and *a*∈ Σ^|*Pa*(ℓ)| ^being a possible observation at the random variables represented by Pa(ℓ) and, hence, corresponding to pa(ℓ, *x*) for a specific sequence *x*.

It follows from these constraints that not all parameters of *θ* are free: if the values of *θ*_1_, *θ*_2_, ..., *θ*_*C*-1 _are given, the value of *θ*_*C *_is determined, and if the values of *θ*_*c*, ℓ, 1, *a*_, *θ*_*c*, ℓ, 2, *a*_, ..., *θ*_*c*, ℓ, *S*-1, *a*_ are given, the value of *θ*_*c*, ℓ, *S*, *a*_ is determined.

### MRF Parametrization of moral Bayesian networks

While generative learning of parameters can be performed analytically for many statistical models, no analytical solution is known for most of the popular models in case of the MCL or the MSP principle. Hence, we must resort to numerical optimization techniques like conjugate gradients or second-order methods [36]. Unfortunately, the parameterization of directed graphical models in terms of *θ* causes two problems in case of numerical optimization: first, the limited domain, which is [0, 1] for probabilities, must be assured, e.g., by barrier methods; second, neither the conditional likelihood  nor its logarithm are concave functions of *θ*, so numerical optimization procedures may get trapped in local maxima or saddle points [27]. Hence, the likelihood of moral Bayesian networks is often defined in an alternative parameterization. We denote these parameters by *λ*which replaces ϑ in equation (2a). This parameterization is closely related to the natural parameters of MRFs [17,40] yielding the likelihood(4)

where *Z *(*λ*) denotes a normalization constant defined as the sum over all possible classes *c *∈  and all possible sequences *x *∈ Σ^*L *^of the numerator:(5)

Similar to the *θ*-parameters, there is one parameter *λ*_*c *_∈ ℝ for each class *c *∈ , and one parameter  ∈ ℝ for each class *c *and each symbol *b *at X_ℓ _given the observation *a *at random variables represented by the nodes Pa(ℓ). In contrast to *θ*, however, these parameters cannot be interpreted directly as probabilities.

As for the *θ*-parameters, not all parameters of *λ* are free. In case of *λ*-parameters, we may fix one of the parameters in each subset, i.e., one of the *λ*_*c *_and one of the *λ*_*c*, ℓ, *b*, *a*_ for each *c *∈ , ℓ ∈ [1, L], and a∈ Σ^|Pa(ℓ)| ^to a constant value without reducing the codomain of *p*_λ _(*x*, *c*|*λ*), resulting in the same number of free parameters for *θ*and *λ*. We choose to fix the last parameter in each subset arbitrarily to 0, i.e.,

In order to show that equations (3) and (4) are equivalent, we need a bijective mapping from *θ*to *λ*. The mapping from *θ*to *λ*is defined by [41]

with *c *∈ [1, *C *- 1] and

*c *∈ [1, *C*], ℓ ∈ [1, *L*], *b *∈ [1, *S *- 1], *a*∈ Σ^|Pa(ℓ)|^, respectively.  The mapping *t* from *λ*to *θ*is less trivial. We denote by [*t*(*λ*)]_*c *_:= *θ*_*c *_the component of *t*defining *θ*_*c*_, and we denote by [*t*(*λ*)]_*c*, ℓ, *b*, *a*_:= *θ*_*c*, ℓ, *b*, *a*_the component of *t*defining *θ*_*c*, ℓ, *b*, *a*_. Then, we obtain *t*by marginalization of (4):(6a)

and(6b)

where *Z*_*c *_(*λ*) and Z_*c*, ℓ, *b*, *a*_(*λ*) are two partial normalisation constants defined in Appendix A of Additional File 1.

### Prior for moral Bayesian networks

For Bayesian learning principles (equations (1a) and (2a)), we must to specify a prior on the parameters of the model. One conjugate prior *h*_*θ *_(*θ*|*α*) for the likelihood of directed graphical models and their specializations is the product-Dirichlet prior [39]. The product-Dirichlet prior assumes parameter independence and amounts to a product of independent Dirichlet densities:(7a)

where *θ*_*C *_:= (*θ*_1_, *θ*_2_, ..., *θ*_*C*_), *α*_*C *_:= (*α*_1_, *α*_2_, ..., *α*_*C*_), *θ*_*c*, ℓ, *a*_:= (*θ*_*c*, ℓ,1, *a*_, ..., *θ*_*c*, ℓ, *S*, *a*_), *α*_*c*, ℓ, *a*_:= (*α*_*c*, ℓ, 1, *a*_, ..., *α*_*c*, ℓ, *S*, *a*_), and(7b)

where ϕ= (ϕ_1_, ϕ_2_, ...), and ϕ_*i *_stands for *θ*_*c *_or *θ*_*c*, ℓ, *b*, *a*_.

We use hyper-parameters *α*that satisfy the *consistency *condition [35,39], which introduces the following constraints on the hyper-parameters *α*. We assume that there are *joint *hyper-parameters *α*_*c*, x_with *x*∈ Σ^*L *^and *c *∈  such that for all ℓ ∈ [1, *L*], for all *b *∈ Σ, and for all *a*∈ Σ^|Pa(ℓ)|^(8a)

and(8b)

where the Kronecker symbol *δ *is 1 if both indices are equal and 0 otherwise. These constraints ensure that the hyper-parameters *α*of the product-Dirichlet prior can be interpreted as, possibly real-valued, counts stemming from a set of a-priorily observed pseudo-data. The size of the set of pseudo-data is commonly referred to as *equivalent sample size *[35,39], and we denote the equivalent sample size of class *c *by *α*_*c*_. Hence, a, product-Dirichlet prior allows an intuitive and easily-interpretable choice of hyper-parameters, in contrast to product-Gaussian or product-Laplace priors.

Our first goal is to derive a prior for *λ*which is equivalent to the commonly-used product-Dirichlet prior for *θ*in equation (7a). To this end, we use the transformation *t*from *λ*to *θ*to transform the product-Dirichlet prior *h*_*θ *_(*θ*|*α*) to the desired prior,(9)

where det (*t'*(*λ*)) denotes the Jacobian of *t*. We derive the Jacobian in Appendix B of Additional File 1 by exploiting independencies between parameters of the model,(10)

and obtain a general transformed Dirichlet prior (Appendix C of Additional File 1).

If all hyper-parameters are chosen to satisfy the consistency condition, many normalization constants cancel, and we obtain a simplified expression of the transformed Dirichlet prior,(11)

where *α *:= Σ_*c *_*α*_*c*_.

Since the commonly-used product-Dirichlet prior for *θ*defined in equation (7a) is conjugate to the likelihood defined in equation (3), the transformed prior of equation (11) is also conjugate to the likelihood defined in equation (4). While in earlier comparisons of different learning principles for the same moral Bayesian network, different priors have been employed, we are now capable of using the same prior as defined in equation (11) for the MAP and the MSP principle. Employing this prior, we can compare the classification accuracy of two classifiers based on the same model, but trained either by the MAP or the MSP principle, using the same prior, avoiding a potential bias induced by differing priors.

### Choice of hyper-parameters

In contrast to the comparison of the MAP and the MSP principle for the same model, the derived prior cannot be used for an unbiased comparison of different models without further premises, since different models typically use different parameters of potentially different dimension, inevitably leading to different priors for these models. One reasonable requirement for the comparison of models with different graph structures is *likelihood equivalence *[39], stating that models with different graph structures representing the same likelihood, also obtain the same *marginal likelihood *of the data given graph structure and hyper-parameters or, equivalently, that the values of the prior density on the parameters of such models must be equal for equivalent parameter values. Examples for different graph structures representing the same likelihood are left-to-right and right-to-left Markov models or differently rooted Bayesian trees with the same undirected graph structure.

Heckerman et al. [39] show that this property is satisfied only by the *BDe metric*, which corresponds to the consistency condition presented above. This condition also entails that the hyper-parameters used for the priors of these models can be derived from a common set of pseudo-data. However, the consistency criterion does not determine how a specific set of pseudo-data should be chosen in order to minimize the bias imposed on the comparison, and different choices may favor different models in one way or the other. For example, a comparison of different models can be easily biased if the set of pseudo-data contains statistical dependencies that can be exploited by some but not by all models, as for instance dinucleotide dependencies that can be captured by a WAM model but not by a PWM model.

The *BDeu metric *[35,39] is a special case of the BDe metric and a popular choice for structure learning and model selection for Bayesian networks [39,42,43] or Bayesian trees and mixtures thereof [2,41]. It imposes additional constraints on the hyper-parameters, which can be described as follows: building on the consistency condition for the product-Dirichlet prior, the specific hyper-parameters for the priors of different models represent identical sets of pseudo-data. The hyper-parameters, which represent the a-priori information, are defined based on a set of pseudo-data in which all possible sequences *x*∈ Σ^*L *^occur with equal probability [35]. Despite the general assumption of uniform pseudo-data, the equivalent sample size may differ between the different classes *c *∈ , representing a-priori class-probabilities. Using the concept of joint hyper-parameters introduced for the consistency condition in the previous subsection, this a-priori information implies that for each class *c *the joint hyper-parameters *α*_*c*, *x*_ are identical for each *x*. For this reason, we derive from equation (8a)

which implies the following values of the hyper-parameters *α*_*c*,ℓ,*b*, *a*_for the model parameters *λ*_*c*,ℓ,*b*, *a*_

where |Pa(ℓ) | is the number of parents Pa(ℓ) of node ℓ, *c *∈ , ℓ ∈ [1, *L*], *b *∈ Σ, and *a *∈ Σ^|Pa(ℓ)|^.

Consider the example that the equivalent sample size for class *c *is *α*_*c *_= 32 and that the data of each class is modeled either by a PWM or by a WAM model. The PWM model has parameters *λ*_*c*, ℓ, *b*_, ℓ ∈ [1, L], *b *∈ Σ, while the WAM model has parameters , *b *∈ Σ and , ℓ ∈ [2, L], *b*, *a *∈ Σ. In case of the DNA alphabet, the BDeu metric determines the hyper-parameters for the PWM model to be *α*_*c*, ℓ, *b *_= 8, while it determines the hyper-parameters for the WAM model to be  = 8 and  = 2. With this choice of hyper-parameters, both product-Dirichlet priors represent the same set of pseudo-data. The hyper-parameters *α*_*c*, ℓ, *b *_of the PWM model correspond to pseudo-counts of mono-nucleotides *b*, while the hyper-parameters  of the WAM model correspond to conditional pseudo-counts of nucleotides *b *given nucleotide *a *observed at the previous position ℓ - 1. This result does equally hold for all specializations of MRFs considered in this paper, and we choose the hyper-parameters accordingly throughout the case studies.

### Markov random fields

The prior of equation (11) allows an unbiased comparison of different learning principles including the generative MAP principle and the discriminative MSP principle for different models from the family of moral Bayesian networks including PWM models, WAM models, Markov models of higher order, or Bayesian trees. However, several important models proposed for the recognition of short signal sequences do not belong to this family. Hence, we now focus on the main goal of deriving a prior for the family of MRFs, which contains the family of moral Bayesian networks as special case.

MRFs are undirected graphical models, i.e., the underlying graph structure is an undirected graph. Again, edges between nodes model potential statistical dependencies between the random variables represented by these nodes, while the absence of edges between nodes represents conditional independencies of the associated random variables given their neighboring nodes. The likelihood of an MRF in terms of *λ*-parameters is given by(12)

where *I*_*c *_denotes the number of *λ*-parameters conditional on class *c*, and *f*_*c*, *i*_(*x*) ∈ {0, 1} denotes the indicator function of *λ*_*c*, *i *_[17,40]. These indicator functions determine the undirected graph structure.

For illustration purposes, we rewrite the likelihood of a PWM in analogy to the MRF likelihood, for which the set of parents of all nodes are empty. Hence, we omit the vector of parents when rewriting the likelihood of equation (4) in terms of Kronecker symbols *δ*,(13)

Renaming the parameters in terms of *λ*_*c*, *i *_and defining the indicator functions *f*_*c*, *i *_as corresponding Kronecker symbols, we obtain the likelihood in form of equation (12).

Using the conformity of equations (4) and (12), we can now suggest a prior for MRFs in analogy to equation (11),(14)

that contains the transformed Dirichlet prior of equation (11) as special case if the MRF of each class belongs to the family of moral Bayesian networks. Examining the likelihood of equation (12), we find that the prior of equation (14) is conjugate to the likelihood of MRFs. Additionally, it is equivalent to the conjugate prior of the exponential family [44] for the studied family of models.

**Figure 1:**
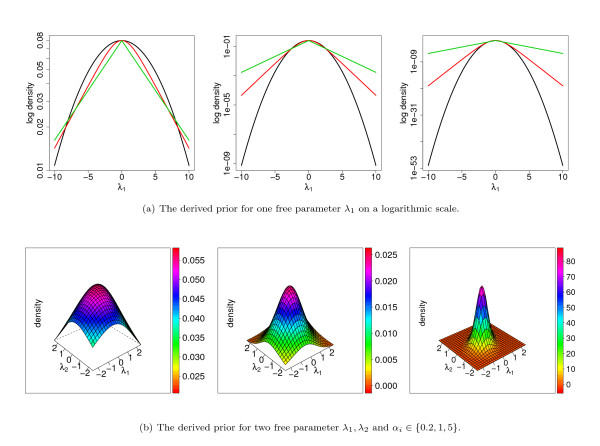
**Illustration of the derived prior**. Illustration of the derived prior of Eqn. (14) for one and two free parameters. Figure a) shows the derived prior (red line) for one free parameter *λ*_1 _and *α*_*i *_∈ {0.2, 1, 5} in comparison to a Gaussian (black line) and a Laplace prior (green line). Figure b) shows the derived prior for two free parameters *λ*_1_, *λ*_2 _and *α*_*i *_∈ {0.2, 1, 5}.

We illustrate the prior of equation (14) for one and two free parameters in Figure 1 for different values of the hyper-parameters *α*_*i*_. In Figure 1a, we compare the derived prior to the Gaussian prior and the Laplace prior for one free parameter *λ*_1_. For illustration purposes, we choose the hyper-parameters of the Gaussian and Laplace prior such that their maxima are identical to that of the derived prior. We find that the derived prior provides an interesting interpolation between a Gaussian prior and a Laplace prior. In the vicinity of the maximum, the logarithm of the derived prior shows a quadratic dependence on *λ*_1_, whereas it shows a linear dependence on *λ*_1 _in the far tails. That is, the derived prior is similar to a Gaussian prior in the vicinity of the maximum and similar to a Laplace prior in the far tails.

In Figure 1b, we show the derived prior for two free parameters *λ*_1 _and *λ*_2_. Interestingly, the derived prior exhibits a mirror symmetry about the plane *λ*_1 _= *λ*_2_, which can be explained by the choice of equal hyper-parameters *α*_1 _= *α*_2_. In contrast to the product-Gaussian and the product-Laplace prior, we do not find a radial symmetry, which can be explained by the fixed parameter *λ*_3 _= 0.

Summarizing the main result of this section, we propose a prior for MRFs that

i) can be used for the generative MAP and the discriminative MSP principle,

ii) is conjugate to the likelihood of MRFs and, hence, also to the likelihoods of many popular models used for the recognition of short sequence motifs,

iii) includes the commonly-used product-Dirichlet prior of equation (7a) as special case if the MRF belongs to the family of moral Bayesian networks including PWM models, WAM models, Markov models of higher order, or Bayesian trees, and

iv) allows to incorporate prior knowledge intuitively by defining a set of a-priorily observed pseudo-data.

Hence, it can be employed in comparative studies of generative and discriminative learning principles applied to the same family of models, and of different, generatively or discriminatively trained models. Additionally, the derived prior can be readily extended to mixtures of models from the family of MRFs. In the next section, we illustrate the utility of the derived prior.

## Results and Discussion

In this section, we present two case studies that illustrate how the derived prior can be used for an unbiased comparison of different learning principles for different models related to two standard problems in bioinformatics.

In case study 1, we illustrate the comparison of different learning principles for the recognition of TFBSs using the same models and the same priors. Specifically, we investigate the influence of different sizes of data sets on the performance of generatively and discriminatively trained models in close analogy to the pioneering study of Ng & Jordan [11]. Possibly due to the lack of a common prior that could be used for both the generative and the discriminative learning, Ng & Jordan compare the generative Bayesian approach of parameter estimation (MAP) to the discriminative non-Bayesian approach of parameter estimation (MCL). Based on the derived prior, it is now possible to compare the two Bayesian learning principles directly using exactly the same prior in both cases. In case of TFBSs, the number of available training sequences is small, typically ranging from only 20 to at most 300 sequences. Hence, available algorithms for the recognition of TFBSs are far from being perfect, and unbiased comparisons of different learning principles for data sets of this size are of fundamental importance for any further advance on this field.

In case study 2, we illustrate the comparison of different learning principles with different models for the recognition of human donor splice sites using the same a-priori information. Donor splice sites exhibit non-adjacent dependencies [3,45,46]. Hence, it seems worthwhile to employ MRFs for this task, as they are capable of capturing dependencies between all pairs of positions in a sequence [5]. However, different subclasses of donor splice sites exist [3], so the use of mixtures of MRFs may be favourable. Donor splice sites are highly conserved so that for some pairs of positions some of the 16 possible pairs of nucleotides do not occur. These non-occurrences cause numerical problems when using the ML or MCL principle, but one may adopt a Bayesian approach to circumvent these problems. Interestingly, mixtures of MRFs have not been employed in the past for the classification of donor splice sites, possibly because of the lack of a suitable prior. The derived prior now provides an opportunity to investigate if mixtures of MRFs might be useful for the recognition of splice sites. We compare mixtures of MRFs to single MRFs, mixtures of Markov models, and single Markov models using the MAP and the MSP principle, and we investigate which of these two learning principles may be worthwhile for the recognition of splice sites.

The focus of the case studies presented is not on the identification of the most appropriate model class or learning principle for the recognition problem scrutinized, although undoubtedly this is a welcome side-effect, but primarily we aim at illustrating the benefit of the derived prior for unbiased comparative studies in bioinformatics.

### Case Study 1: Discriminative vs. generative parameter estimation

In case study 1, we illustrate a comparison of generatively trained and discriminatively trained Markov models of different orders using the derived prior. We choose the data set of [26] containing 257 aligned binding sites, each of length 16 bp, of the mammalian transcription factor Sp1 as foreground data set and 267 second exons of human genes, which have different lengths and are cut into 100-mers for this study, with a total size of approximately 68 kb as background data set. We use a PWM model as foreground model and Markov models of order 3 as background model. Results for all other combinations of a Markov model of orders 0 or 1 as foreground model and Markov models of orders 0 to 3 as background model are available in Additional File 2. These models are trained by the MAP principle and by the MSP principle using the same priors and the same hyper-parameters for both cases. We choose for both cases and all model combinations an equivalent sample size of 4 for the foreground model and an equivalent sample size of 1024 for the background model.

We use a *stratified holdout sampling *procedure for the comparison of the classification performance of the resulting classifiers. In each iteration of the stratified holdout sampling procedure, we randomly partition both the foreground data set and the background data set into a preliminary training data set comprising 90% of the sequences and a test data set comprising the remaining 10% of the sequences. In order to vary the size of the training data set, we use an additional sampling step, where we randomly draw a given fraction of the preliminary training data sets ranging from 5% to 100% yielding the final training data sets. We train all classifiers corresponding to different learning principles and different model combinations on the same subsets of the preliminary training data sets, and we use the resulting classifiers to classify the same sequences in the test data sets.

**Figure 2:**
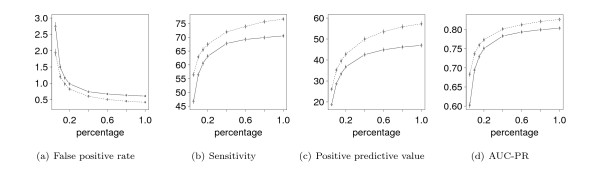
**Comparing generatively to discriminatively trained models**. We compare the classification performance of classifiers using the MAP principle (solid line) and the MSP principle (dashed line) with the derived prior on differently-sized training data sets for binding sites of the transcription factor Sp1. For both classifiers, we use a PWM model in the foreground and a Markov model of order 3 in the background. We plot the four performance measures, false positive rate, sensitivity, positive predictive value, and area under the precision-recall curve (AUC-PR), against the percentage of the preliminary training data set used for estimating the parameters. Whiskers indicate two-fold standard errors. We find that the classification performance increases with increasing size of the training data set. For the false positive rate this corresponds to a decreasing curve. For all four measures and all sizes of the data set, we find that the discriminatively trained Markov models yield a consistently higher classification performance than the generatively trained Markov models.

We evaluate the classification performance on the test data sets using as performance measures the false positive rate (FPR) for a fixed sensitivity of 95%, the sensitivity (Sn) for a fixed specificity of 99.9%, the positive predictive value (PPV) for a fixed sensitivity of 95%, and the area under the precision recall curve (AUC-PR) [26,47]. We repeat the stratified holdout sampling procedure 1, 000 times, and report the means and standard errors of the four performance measures FPR, Sn, PPV, and AUC-PR for each classifier as the final result of the comparison. We present the results of the comparison for the combination of a PWM model in the foreground and a Markov model of order 3 in the background in Figure 2, which shows the four performance measures Sn, FPR, PPV, and AUC-PR as functions of the relative size of the training data sets. Corresponding results for other combinations of models show the same qualitative behaviour and are available in Additional File 2.

The classification performance increases rapidly with increasing size of the training data set and achieves its optimal value for the largest training data sets. For the largest training data set, the discriminatively trained classifier yields an FPR of 0.4%, an Sn of 76.6%, a PPV of 57.3%, and an AUC-PR of 0.826, whereas the generatively trained classifier yields only an FPR of 0.6%, an Sn of 70.5%, a PPV of 47.0%, and an AUC-PR of 0.803.

Ng & Jordan [11] compare the classification performance of PWMs trained by the MAP principle and the MCL principle on a number of data sets from the UCI machine learning repository. They find that for large data sets the discriminative MCL principle has a lower asymptotic error, corresponding to a higher classification performance, but that the generative MAP principle yields a higher classification performance for small data sets. In contrast to those findings, we find a superior classification performance of the discriminatively compared to the generatively trained models irrespective of the size of the training data set. This result suggests that the choice of the same prior is advisable for an unbiased comparison of generative and discriminative learning principles and, moreover, that it might be worthwhile to re-evaluate the power of the MSP principle for other applications in bioinformatics as well.

### Case Study 2: Mixtures of Markov random fields

In this case study, we demonstrate a comparison of different learning principles using Markov models, mixtures of Markov models, MRFs, and mixtures of MRFs, and the derived prior. We choose a standard data set of human donor splice sites (foreground data set) and human non-donor splice sites (background data set) compiled by Yeo & Burge [5]. This data set is already partitioned into a foreground training data set (8, 415 donor splice sites), a background training data set (179, 438 non-splice sites), a foreground test data set (4, 208 donor splice sites), and a background test data set (89, 717 non-splice sites). We choose an inhomogeneous Markov model of order 1 (MM) and an MRF which models all pairwise dependencies [5] as basic models. The MRF has 336 indicator functions each of the form(15)

where ℓ_1_, ℓ_2 _∈ [1, *L*], ℓ_1 _≠ ℓ_2_, and b_1_, b_2 _∈ Σ. Based on these basic models, we build mixture models with two MMs (mixMM) and two such MRFs (mixMRF), and we compare those four classifiers that are based on a combination of the same kind of model for the foreground and for the background class. For all of these classifiers, we use the derived prior with an equivalent sample size of 32 for each of the four foreground models and an equivalent sample size of 96 for each of the four background models. We train each of these classifiers on the two training data sets using the MAP and the MSP principle, and we evaluate their classification performance on the two test data sets. We use the same performance measures as in case study 1, except that we replace Sn by the the area under the receiver operating characteristic curve (AUC-ROC) [48], because AUC-ROC is more commonly used than Sn for the classification of splice sites [5].

**Figure 3:**
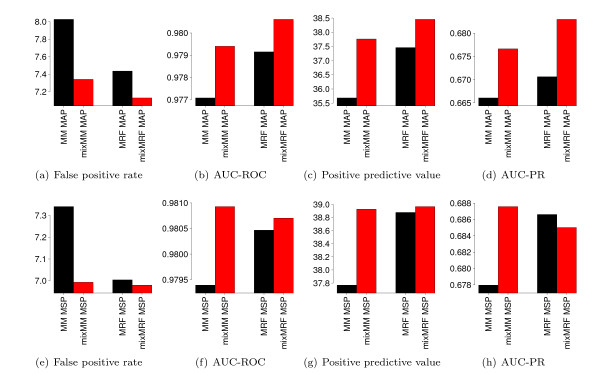
**Comparison of different generatively and discriminatively trained models**. We compare the classification performance of Markov models (MM), mixtures of Markov models (mixMM), Markov random fields (MRF), and mixtures of Markov random fields (mixMRF) for a set of donor splice sites [5] using the MAP and the MSP principle, and using the derived prior for all models. We plot the four performance measures false positive rate, area under the ROC curve (AUC-ROC), positive predictive value, and area under the precision-recall curve (AUC-PR) for each of the four models. For the MAP principle (a-d), the comparison shows that mixMM and mixMRF yield a higher classification performance than MM and MRF, respectively, and that mixMRF achieves the highest classification performance of all models with respect to all four performance measures. For the MSP principle (e-h), the comparison shows that mixMM and mixMRF yield a higher classification performance than MM and MRF, respectively, and that mixMRF achieves the highest classification performance of all models with respect to false positive rate and positive predictive value, whereas the highest AUC-PR and AUC-ROC are achieved by mixMM.

We present the results of this comparison in Figure 3, which shows barplots of each of the four performance measures for each of the four classifiers and both learning principles. The results for the MAP principle are shown in Figure 3(a-d). We find that the two classifiers based on mixture models outperform the two corresponding classifiers based on single models with respect to all four performance measures. We also find that the two classifiers based on MRFs and mixMRFs yield a higher classification performance than the two corresponding classifier based on MMs and mixMMs. The classifier based on a mixture of MRFs yields the lowest FPR (7.1%), the highest AUC-ROC (0.9806), the highest PPV (38.5%), and the highest AUC-PR (0.6830), stating that, among the four models tested, a mixMRF is the most appropriate model for classifying human donor splice sites and non-donor sites using the MAP principle.

In close analogy to Figure 3(a-d), (e-h) shows the results using the MSP principle. We find that discriminatively trained mixture models, i.e., mixMM and mixMRF, outperform the two corresponding classifiers based on the single MM and single MRF, and that the mixMM classifier is comparable or even better than the MRF classifier. The mixMRF classifier yields the best results for FPR (7.0%) and PPV (39.0%), while the mixMM classifier yields a higher AUC-ROC (0.9809) and AUC-PR (0.6876) than the mixMRF classifier.

Comparing Figures 3(a-d) and 3(e-h), we find that the four MSP-trained models outperform the corresponding MAP-trained models. For instance, the MM classifier yields an PPV of 37.8% for the MSP principle and only 35.7% for the MAP principle, and the mixMRF classifier yields a PPV of 39.0% for the MSP principle only 38.5% for the MAP principle. Interestingly, classifiers based on simple models (MM and mixMM) show the greatest improvement when replacing the MAP principle by the MSP principle. This observation is in accordance with previous findings that discriminative learning seems to be advantageous over generative learning if the model assumption is wrong [29].

## Conclusions

The systematic comparison of different statistical models and different learning principles has been the focus of several studies of the last decade [11,26,29,30]. However, these comparisons lose value if different priors are used for different models or different learning principles, and it is questionable if the obtained results from such comparisons are meaningful at all.

In this paper, we derive a prior that allows an unbiased comparison of generative and discriminative learning principles for models from the family of MRFs including PWM models, WAM models, Markov models of higher order, Bayesian trees, moral Bayesian networks, and their mixtures as special cases. The derived prior is conjugate to the likelihood of MRFs and a generalization of the commonly-used product-Dirichlet prior for moral Bayesian networks. The derived prior provides an interesting interpolation between a product-Gaussian prior and a product-Laplace prior: it is qualitatively similar to a product-Gaussian prior in the vicinity of the maximum and qualitatively similar to a product-Laplace prior in the far tails. In contrast to a product-Gaussian and a product-Laplace prior, the hyper-parameters of the derived prior can be easily interpreted as counts stemming from pseudo-data, allowing an intuitive choice of these hyper-parameters.

We present two case studies using the derived prior for an unbiased comparison, and we find that discriminative parameter learning can be beneficial for sequence classification in the field of bioinformatics. On a set of mammalian TFBSs, we find that it is possible to yield an improved classification performance by using the discriminative MSP principle instead of the generative MAP principle even if the amount of available training data is small. By varying the size of the training data set, we find that discriminative parameter learning can improve the recognition of TFBSs over generative parameter learning irrespective of the size of the training data set. This result differs from previous findings of Ng & Jordan [11], who did a similar study comparing the generative Bayesian MAP principle to the discriminative non-Bayesian MCL principle. On a data set of donor splice sites [5], we illustrate the utility of the proposed prior for comparing Markov models, mixtures of Markov models, MRFs, and mixtures of MRFs. For this data set, we find that the best classification performance can be achieved by a discriminatively trained mixture of MRFs.

The derived prior might be useful in future comparative studies as it provides a less-biased guidance to the understanding of molecular mechanisms, and it leads to further improvements of algorithms for the recognition of short signal sequences including splice sites, TFBSs, nucleosome binding sites, miRNA binding sites, transcription initiation sites, or insulator binding sites. Hence, we make an implementation of this prior available to the scientific community as part of the open source Java library Jstacs http://www.jstacs.de.

## List of abbreviations

MAP: maximum a-posteriori; MCL: maximum conditional likelihood; ML: maximum likelihood; MRF: Markov random field; MSP: maximum supervised posterior; PWM: position weight matrix; TFBS: transcription factor binding sites; WAM: weight array matrix.

## Authors' contributions

IG and JK developed the basic idea. JK and JG derived the prior, implemented the software, and performed the case studies. All authors contributed to writing and approved the final manuscript.

## Supplementary Material

Additional file 1 **Appendices**. This file contains more information about the partial normalization constants, the computation of the Jacobian, and a general prior for moral Bayesian networks. Click here for file

Additional file 2 **Results of the Sp1 case study**. This file contains all results of the Sp1 case study including for all combinations of Markov models. For the foreground class we use orders 0 or 1, and for the background class we use orders 0 to 3. Click here for file

## References

[B1] Kel AE Gössling E Reuter I Cheremushkin E Kel-Margoulis OV Wingender E MATCH: A tool for searching transcription factor binding sites in DNA sequences Nucleic Acids Res 2003 31 13 3576 3579 10.1093/nar/gkg585 12824369 PMC169193

[B2] Barash Y Elidan G Friedman N Kaplan T Modelling dependencies in protein-DNA binding sites RECOMB '03: Proceedings of the seventh annual international conference on Research in computational molecular biology 2003 New York, NY, USA: ACM Press 28 37 full_text

[B3] Burge C Karlin S Prediction of complete gene structures in human genomic DNA Journal of Molecular Biology 1997 268 78 94 10.1006/jmbi.1997.0951 9149143

[B4] Salzberg SL A method for identifying splice sites and translational start sites in eukaryotic mRNA Comput Appl Biosci 1997 13 4 365 376 9283751 10.1093/bioinformatics/13.4.365

[B5] Yeo G Burge CB Maximum Entropy Modeling of Short Sequence Motifs with Applications to RNA Splicing Signals Journal of Computational Biology 2004 11 2-3 377 394 10.1089/1066527041410418 15285897

[B6] Segal E Fondufe-Mittendorf Y Chen L Thåström A Field Y Moore IK Wang JPZ Widom J A genomic code for nucleosome positioning Nature 2006 442 7104 772 778 10.1038/nature04979 16862119 PMC2623244

[B7] Peckham HE Thurman RE Fu Y Stamatoyannopoulos JA Noble WS Struhl K Weng Z Nucleosome positioning signals in genomic DNA Genome Res 2007 gr.6101007+ 17620451 10.1101/gr.6101007 PMC1933512

[B8] Kim TH Abdullaev ZK Smith AD Ching KA Loukinov DI Green RD Zhang MQ Lobanenkov VV Ren B Analysis of the vertebrate insulator protein CTCF-binding sites in the human genome Cell 2007 128 6 1231 1245 10.1016/j.cell.2006.12.048 17382889 PMC2572726

[B9] Redhead E Bailey T Discriminative motif discovery in DNA and protein sequences using the DEME algorithm BMC Bioinformatics 2007 8 385 10.1186/1471-2105-8-385 17937785 PMC2194741

[B10] Tompa M Li N Bailey TL Church GM De Moor B Eskin E Favorov AV Frith MC Fu Y Kent WJ Makeev VJ Mironov AA Noble WS Pavesi G Pesole G Regnier M Simonis N Sinha S Thijs G van Helden J Vandenbogaert M Weng Z Workman C Ye C Zhu Z Assessing computational tools for the discovery of transcription factor binding sites Nat Biotech 2005 23 137 144 10.1038/nbt1053 15637633

[B11] Ng AY Jordan MI Dietterich T, Becker S, Ghahramani Z On discriminative vs. generative classifiers: A comparison of logistic regression and naive bayes Advances in Neural Information Processing Systems 2002 14 Cambridge, MA: MIT Press 605 610

[B12] Ben-Gal I Shani A Gohr A Grau J Arviv S Shmilovici A Posch S Grosse I Identification of transcription factor binding sites with variable-order Bayesian networks Bioinformatics 2005 21 11 2657 2666 10.1093/bioinformatics/bti410 15797905

[B13] Sonnenburg S Zien A Rätsch G ARTS: accurate recognition of transcription starts in human Bioinformatics 2006 22 14 e472 480 10.1093/bioinformatics/btl250 16873509

[B14] Kim NK Tharakaraman K Marino-Ramirez L Spouge J Finding sequence motifs with Bayesian models incorporating positional information: an application to transcription factor binding sites BMC Bioinformatics 2008 9 262 10.1186/1471-2105-9-262 18533028 PMC2432075

[B15] Narlikar L Gordan R Ohler U Hartemink AJ Informative priors based on transcription factor structural class improve de novo motif discovery Bioinformatics 2006 22 14 e384 392 10.1093/bioinformatics/btl251 16873497

[B16] Chen S Rosenfeld R A Gaussion Prior for Smoothing Maximum Entropy Models Tech. rep., School of Computer Science 1999 Carnegie Mellon University, Pittsburgh, PA

[B17] Klein D Manning C Maxent Models, Conditional Estimation, and Optimization HLT-NAACL 2003 Tutorial 2003

[B18] Staden R Computer methods to locate signals in nucleic acid sequences Nucleic Acids Research 1984 12 505 519 10.1093/nar/12.1Part2.505 6364039 PMC321067

[B19] Stormo GD Schneider TD Gold LM Ehrenfeucht A Use of the 'perceptron' algorithm to distinguish translational initiation sites NAR 1982 10 2997 3010 10.1093/nar/10.9.2997 7048259 PMC320670

[B20] Zhang M Marr T A weight array method for splicing signal analysis Comput Appl Biosci 1993 9 5 499 509 8293321 10.1093/bioinformatics/9.5.499

[B21] Yakhnenko O Silvescu A Honavar V Discriminatively Trained Markov Model for Sequence Classification ICDM '05: Proceedings of the Fifth IEEE International Conference on Data Mining, Washington, DC, USA: IEEE Computer Society 2005 498 505 full_text

[B22] Keilwagen J Grau J Posch S Grosse I Hinneburg A Recognition of splice sites using maximum conditional likelihood LWA: Lernen - Wissen - Abstraktion 2007 67 72

[B23] Cai D Delcher A Kao B Kasif S Modeling splice sites with Bayes networks Bioinformatics 2000 16 2 152 158 10.1093/bioinformatics/16.2.152 10842737

[B24] Culotta A Kulp D McCallum A Gene Prediction with Conditional Random Fields Tech. Rep. Technical Report UM-CS-2005-028 2005 University of Massachusetts, Amherst

[B25] Bernal A Crammer K Hatzigeorgiou A Pereira F Global Discriminative Learning for Higher-Accuracy Computational Gene Prediction PLoS Comput Biol 2007 3 3 e54 10.1371/journal.pcbi.0030054 17367206 PMC1828702

[B26] Grau J Keilwagen J Kel A Grosse I Posch S Falter C, Schliep A, Selbig J, Vingron M, Walter D Supervised posteriors for DNA-motif classification German Conference on Bioinformatics, Volume 115 of Lecture Notes in Informatics (LNI) - Proceedings 2007 Bonn: Gesellschaft für Informatik (GI) 123 134

[B27] Wettig H Grünwald P Roos T Myllymäki P Tirri H On Supervised Learning of Bayesian Network Parameters Tech. Rep. HIIT Technical Report 2002-1, Helsinki Institute for Information Technology HIIT 2002

[B28] Grossman D Domingos P Learning Bayesian network classifiers by maximizing conditional likelihood 2004 ICML, ACM Press 361 368

[B29] Greiner R Su X Shen B Zhou W Structural Extension to Logistic Regression: Discriminative Parameter Learning of Belief Net Classifiers Machine Learning Journal 2005 59 3 297 322 10.1007/s10994-005-0469-0

[B30] Pernkopf F Bilmes JA Discriminative versus generative parameter and structure learning of Bayesian network classifiers Proceedings of the 22nd International Conference on Machine Learning 2005 657 664 full_text

[B31] Feelders A Ivanovs J Discriminative Scoring of Bayesian Network Classifiers: a Comparative Study Proceedings of the third European workshop on probabilistic graphical models 2006 75 82

[B32] Grünwald P Kontkanen P Myllymäki P Roos T Tirri H Wettig H Supervised posterior distributions Presented at the Seventh Valencia International Meeting on Bayesian Statistics 2002

[B33] Cerquides J de Mántaras RL Robust Bayesian Linear Classifier Ensembles ECML 2005 72 83

[B34] Goodman J Exponential Priors for Maximum Entropy Models Proceedings of HLTNAACL 2004 2003

[B35] Buntine WL Theory Refinement of Bayesian Networks Uncertainty in Artificial Intelligence, Morgan Kaufmann 1991 52 62

[B36] Wallach H Efficient Training of Conditional Random Fields Master's thesis 2002 University of Edinburgh

[B37] Jordan MI Graphical Models Statistical Science (Special Issue on Bayesian Statistics) 2004 19 140 155

[B38] Castelo R The discrete acyclic digraph Markov model in data mining PhD thesis 2002 Faculteit Wiskunde en Informatica, Universiteit Utrecht

[B39] Heckerman D Geiger D Chickering DM Learning Bayesian networks: The combination of knowledge and statistical data Machine Learning 1995 197 243

[B40] Berger AL Pietra SD Pietra VJD A Maximum Entropy Approach to Natural Language Processing Computational Linguistics 1996 22 39 71

[B41] Meila-Predoviciu M Learning with Mixtures of Trees PhD thesis 1999 Massachusetts Institute of Technology

[B42] Castelo R Guigo R Splice site identification by idlBNs Bioinformatics 2004 20 suppl_1 i69 76 10.1093/bioinformatics/bth932 15262783

[B43] Schulte O Frigo G Greiner R Luo W Khosravi H A new hybrid method for Bayesian network learning With dependency constraints Bioinformatics 2009 53 60

[B44] Bishop CM Pattern Recognition and Machine Learning 2006 1 Information Science and Statistics, New York: Springer

[B45] Arita M Tsuda K Asai K Modeling splicing sites with pairwise correlations Bioinformatics 2002 18 suppl_2 S27 34 12385980 10.1093/bioinformatics/18.suppl_2.s27

[B46] Chen TM Lu CC Li WH Prediction of splice sites with dependency graphs and their expanded bayesian networks Bioinformatics 2005 21 4 471 482 10.1093/bioinformatics/bti025 15374869

[B47] Davis J Goadrich M The relationship between Precision-Recall and ROC curves ICML '06: Proceedings of the 23rd international conference on Machine learning 2006 New York, NY, USA: ACM 233 240 full_text

[B48] Fawcett T ROC Graphs: Notes and Practical Considerations for Researchers Tech. rep., HP Laboratories 2004

