# Supplementary material for: Apples and oranges: avoiding different priors in Bayesian DNA sequence analysis
Source: BMC Bioinformatics. 2010 Mar 22;11:149. doi: 10.1186/1471-2105-11-149 (PMC2859755; doi:10.1186/1471-2105-11-149)
Supplement: Additional file 1 — Appendices. This file contains more information about the partial normalization constants, the computation of the Jacobian, and a general prior for moral Bayesian networks. [file 1471-2105-11-149-S1.PDF]

Appendix A Partial normalization constants

In section *Methods*, we show in equations (6a) and (6b) how to transform the parameters $\underline{\lambda}$ to parameters $\underline{\theta}$. In this transformation, we use two different types of partial normalization constants. First, we use a partial normalization constant for the classes that sums over all possible observations given the class:

$$Z_c\left(\underline{\lambda}\right) := \sum_{\underline{x}} \exp\left(\sum_{\ell=1}^{L} \lambda_{c,\ell,x_\ell,\underline{\mathrm{pa}}(\ell,\underline{x})}\right). \tag{A.1}$$

Second, we use partial normalization constants $Z_{c,\ell,b,\underline{a}}\left(\underline{\lambda}\right)$ for each node ℓ of the DAG of each class c. These partial normalization constants are defined in close analogy to the previous one by summing over all possible realizations of the random variables in the subgraph under node ℓ given the observation $X_\ell = b$ and given the observations $\underline{\mathrm{Pa}}\left(\ell\right) = \underline{a}$. For shortness of notation, we define the partial transformation constants recursively. If node ℓ of the DAG of class c is a leaf, we define

$$Z_{c,\ell,b,\underline{a}}\left(\underline{\lambda}\right) := 1. \tag{A.2a}$$

Otherwise, following directly from the definition of a moral graph, node ℓ is parent of at least one other node k whose parents are ℓ and a subset of $\underline{\mathrm{Pa}}\left(\ell\right)$. We denote this non-empty set of nodes by K. For each node $k \in K$ the realization of its parent nodes can be obtained directly from b and $\underline{a}$. We define a specific selection function $s_k(b, \underline{a})$ that returns the realizations of $\underline{\mathrm{Pa}}\left(k\right)$ given b and $\underline{a}$. The partial normalization constant is then defined by:

$$Z_{c,\ell,b,\underline{a}}\left(\underline{\lambda}\right) := \sum_{k\in K}\sum_{d\in\Sigma} \exp\left(\lambda_{c,k,d,s_k(b,\underline{a})}\right) \cdot Z_{c,k,d,s_k(b,\underline{a})}\left(\underline{\lambda}\right). \tag{A.2b}$$

Appendix B Determinant of the Jacobian matrix for moral Bayesian networks

In this section, we show the essential steps for the computation of the determinant of the Jacobian matrix required in section *Methods*. The order of the parameters in the parameter vector has no influence on the absolute value of the determinant of the Jacobian matrix, so we choose an ordering which simplifies the further computation. The evident ordering of the parameters, which contains the class parameters at the beginning of the vector followed by the parameters of each class ordered according to the topological ordering of the corresponding nodes, already has this simplifying property. Using this ordering, for a parameter at position k of the parameter vector almost only parameters at positions greater than k in the parameter vector are potentially needed for the transformation from λ to θ (see Eqn. (6a), (6b), (A.2a), and (A.2b)). For this reason, we obtain zero-valued entries for almost all entries of the Jacobian matrix below the diagonal. The only non-zero entries below the diagonal are located in on-diagonal blocks. In the following, we manipulate the Jacobian matrix and especially the on-diagonal blocks to obtain an upper triangular matrix, for which the determinant is simply the product of the diagonal elements implying that all non-diagonal elements are not relevant. Here, we consider the first on-diagonal block for the class parameters $\lambda_1, \ldots, \lambda_{C-1}$ and the fixed parameter λ_C.

For the further derivation, we repeat the transformation of these parameters as stated in Eqn. (6a)

$$\theta_c = \left[\underline{t}(\underline{\lambda})\right]_c = \frac{\exp\left(\lambda_c\right) Z_c\left(\underline{\lambda}\right)}{Z\left(\underline{\lambda}\right)}$$

We consider the partial derivatives of $C-1$ parameters that build the first on-diagonal block $B(\underline{\lambda})$ of the Jacobian matrix:

$$\frac{\partial\left[\underline{t}(\underline{\lambda})\right]_c}{\partial\lambda_j} = \frac{\exp\left(\lambda_c\right) Z_c\left(\underline{\lambda}\right)}{Z\left(\underline{\lambda}\right)^2} \cdot \begin{cases} Z\left(\underline{\lambda}\right) - \exp\left(\lambda_c\right) Z_c\left(\underline{\lambda}\right) & , c = j \\ -\exp\left(\lambda_j\right) Z_j\left(\underline{\lambda}\right) & , c \neq j \end{cases} \tag{B.1}$$

and

$$|\det B(\underline{\lambda})| = \prod_{c=1}^{C-1} \frac{\exp(\lambda_c)\, Z_c(\underline{\lambda})}{Z(\underline{\lambda})^2} \cdot \left| \det \begin{pmatrix} Z(\underline{\lambda}) - \exp(\lambda_1) Z_1(\underline{\lambda}) & -\exp(\lambda_2) Z_2(\underline{\lambda}) & \dots & -\exp(\lambda_{C-1}) Z_{C-1}(\underline{\lambda}) \\ -\exp(\lambda_1) Z_1(\underline{\lambda}) & Z(\underline{\lambda}) - \exp(\lambda_2) Z_2(\underline{\lambda}) & \dots & -\exp(\lambda_{C-1}) Z_{C-1}(\underline{\lambda}) \\ \vdots & \vdots & \ddots & \vdots \\ -\exp(\lambda_1) Z_1(\underline{\lambda}) & -\exp(\lambda_2) Z_2(\underline{\lambda}) & \dots & Z(\underline{\lambda}) - \exp(\lambda_{C-1}) Z_{C-1}(\underline{\lambda}) \end{pmatrix} \right|. \tag{B.2}$$

We compute the determinant in two steps, which are performed on the complete Jacobian matrix. Since the results for all elements that are not located in on-diagonal blocks do not influence the determinant, we do not show these elements and results here.

First, we subtract the first row from all other rows. Second, we add to the first column all the other columns. We obtain an upper triangular block with $\exp(\lambda_C)\, Z_C(\underline{\lambda})$ as first diagonal element and $Z(\underline{\lambda})$ for all $C-2$ other diagonal elements:

$$|\det B(\underline{\lambda})| = \prod_{c=1}^{C-1} \frac{\exp(\lambda_c)\, Z_c(\underline{\lambda})}{Z(\underline{\lambda})^2} \cdot \left| \det \begin{pmatrix} \exp(\lambda_C) Z_C(\underline{\lambda}) & -\exp(\lambda_2) Z_2(\underline{\lambda}) & \dots & -\exp(\lambda_{C-1}) Z_{C-1}(\underline{\lambda}) \\ 0 & Z(\underline{\lambda}) & \dots & 0 \\ \vdots & \vdots & \ddots & \vdots \\ 0 & 0 & \dots & Z(\underline{\lambda}) \end{pmatrix} \right|. \tag{B.3}$$

The determinant can now be computed as the product of the diagonal elements and we obtain

$$|\det B(\underline{\lambda})| = \prod_{c=1}^{C} \frac{\exp(\lambda_c)\, Z_c(\underline{\lambda})}{Z(\underline{\lambda})}. \tag{B.4}$$

We apply these steps to all other on-diagonal blocks $B_{c,\ell,\underline{a}}(\underline{\lambda})$ and obtain

$$|\det B_{c,\ell,\underline{a}}(\underline{\lambda})| = \prod_{b\in\Sigma} \frac{\exp\left(\lambda_{c,\ell,b,\underline{a}}\right) Z_{c,\ell,b,\underline{a}}(\underline{\lambda})}{\sum_{\tilde{b}} \exp\left(\lambda_{c,\ell,\tilde{b},\underline{a}}\right) Z_{c,\ell,\tilde{b},\underline{a}}(\underline{\lambda})}. \tag{B.5}$$

Using equations (B.4) and (B.5), the determinant of the Jacobian matrix is:

$$|\det \underline{t}'(\underline{\lambda})| = \prod_{c\in\mathcal{C}} \frac{\exp(\lambda_c)\, Z_c(\underline{\lambda})}{Z(\underline{\lambda})} \prod_{\ell=1}^{L} \prod_{\underline{a}} \prod_{b\in\Sigma} \frac{\exp\left(\lambda_{c,\ell,b,\underline{a}}\right) Z_{c,\ell,b,\underline{a}}(\underline{\lambda})}{\sum_{\tilde{b}} \exp\left(\lambda_{c,\ell,\tilde{b},\underline{a}}\right) Z_{c,\ell,\tilde{b},\underline{a}}(\underline{\lambda})}. \tag{B.6}$$

Appendix C General prior for moral Bayesian networks

Using the equations 9 and (B.6) which is the result of Appendix B, we obtain

$$h_\lambda(\underline{\lambda}|\underline{\alpha}) = \mathrm{Dit}(\underline{\lambda}_{\mathcal{C}}|\underline{\alpha}_{\mathcal{C}}) \cdot \prod_{c} \prod_{\ell} \prod_{\underline{a}\in\Sigma^{|\mathrm{\underline{Pa}}(\ell)|}} \mathrm{Dit}\left(\underline{\lambda}_{c,\ell,\underline{a}}|\underline{\alpha}_{c,\ell,\underline{a}}\right), \tag{C.1a}$$

where $\underline{\lambda}_{\mathcal{C}} := (\lambda_1, \lambda_2, \dots, \lambda_C)$, $\underline{\lambda}_{c,\ell,\underline{a}} := (\lambda_{c,\ell,1,\underline{a}}, \lambda_{c,\ell,2,\underline{a}}, \dots, \lambda_{c,\ell,S,\underline{a}})$ and

$$\mathrm{Dit}\left(\underline{\psi}|\underline{\alpha}\right) = \frac{\Gamma\left(\sum_i \alpha_i\right)}{\prod_i \Gamma(\alpha_i)} \cdot \frac{\prod_i \left(\exp(\psi_i) \cdot Z_i(\underline{\lambda})\right)^{\alpha_i}}{\left(\sum_i \exp(\psi_i) \cdot Z_i(\underline{\lambda})\right)^{\sum_i \alpha_i}}. \tag{C.1b}$$

where $\underline{\psi}$ is the vector of ψ_i, ψ_i stands for λ_c or $\lambda_{c,\ell,b,\underline{a}}$, and $Z_i(\underline{\lambda})$ denotes $Z_c(\underline{\lambda})$ or $Z_{c,\ell,b,\underline{a}}(\underline{\lambda})$, respectively.
