# Supplementary material for: Apples and oranges: avoiding different priors in Bayesian DNA sequence analysis
Source: BMC Bioinformatics. 2010 Mar 22;11:149. doi: 10.1186/1471-2105-11-149 (PMC2859755; doi:10.1186/1471-2105-11-149)
Supplement: Additional file 2 — Results of the Sp1 case study. This file contains all results of the Sp1 case study including for all combinations of Markov models. For the foreground class we use orders 0 or 1, and for the background class we use orders 0 to 3. [file 1471-2105-11-149-S2.PDF]

(a)-(d) background order 0

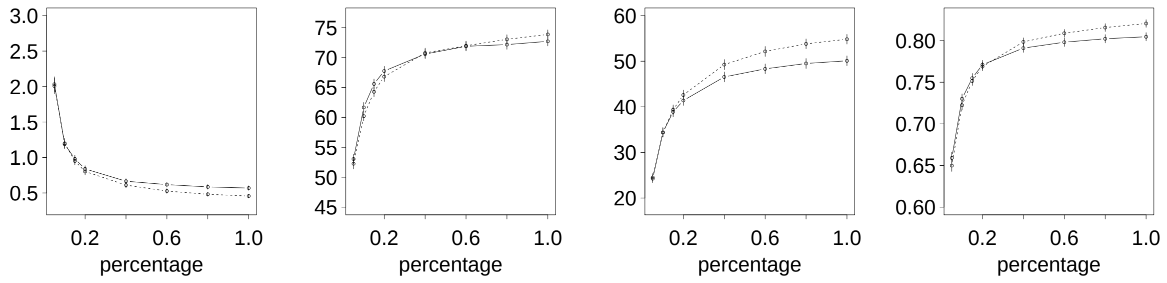

(a) False positive rate (b) Sensitivity (c) Positive predictive value (d) AUC-PR

(e)-(h) background order 1

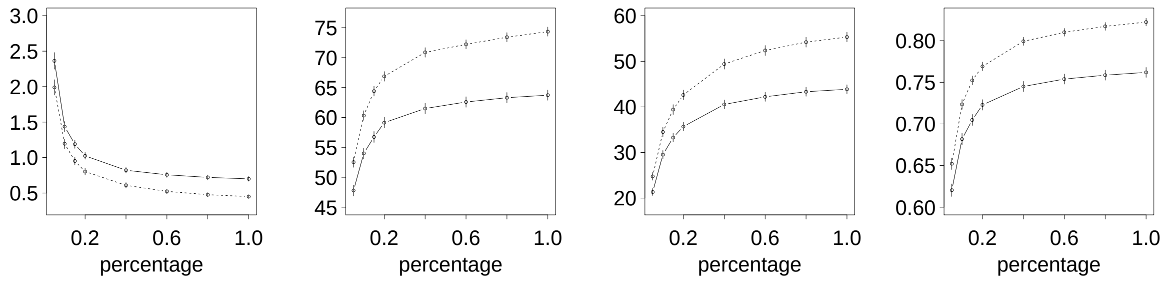

(e) False positive rate (f) Sensitivity (g) Positive predictive value (h) AUC-PR

(i)-(l) background order 2

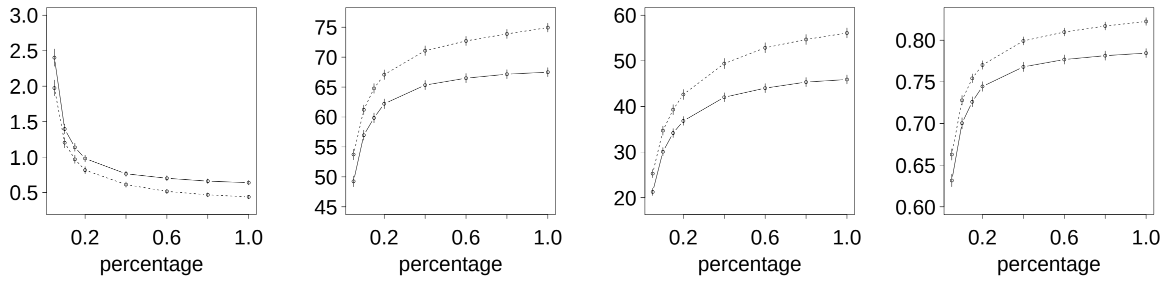

(i) False positive rate (j) Sensitivity (k) Positive predictive value (l) AUC-PR

(m)-(p) background order 3

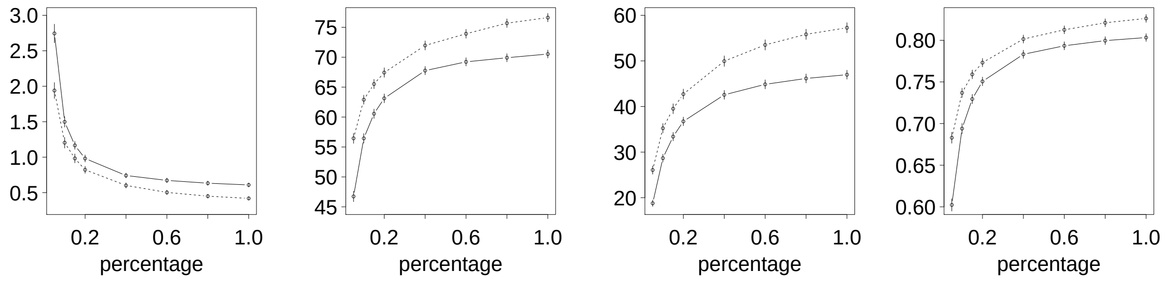

(m) False positive rate (n) Sensitivity (o) Positive predictive value (p) AUC-PR

Figure 1: Classification performance of generatively (solid line) and discriminatively (dashed line) trained Markov models on differently sized training data sets and different background orders for binding sites of the Sp1 transcription factor. We use a Markov model of order 0 in the foreground and Markov models of varying order in the background. We plot the four performance measures, false positive rate, sensitivity, positive predictive value, and area under the precision-recall curve (AUC-PR) against the percentage of the training data set used to estimate the parameters.

(a)-(d) background order 0

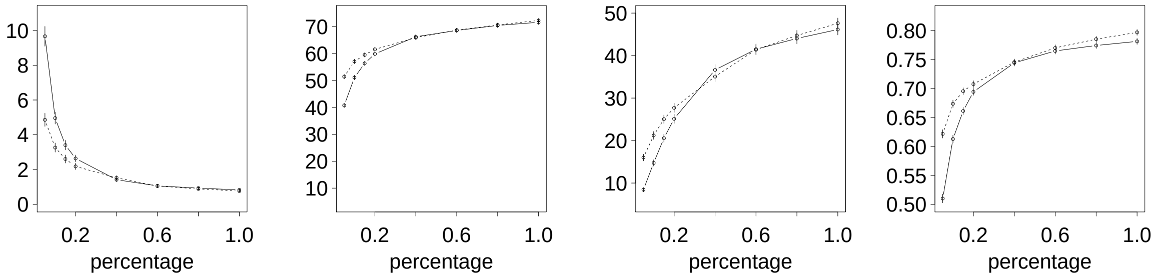

(a) False positive rate (b) Sensitivity (c) Positive predictive value (d) AUC-PR

(e)-(h) background order 1

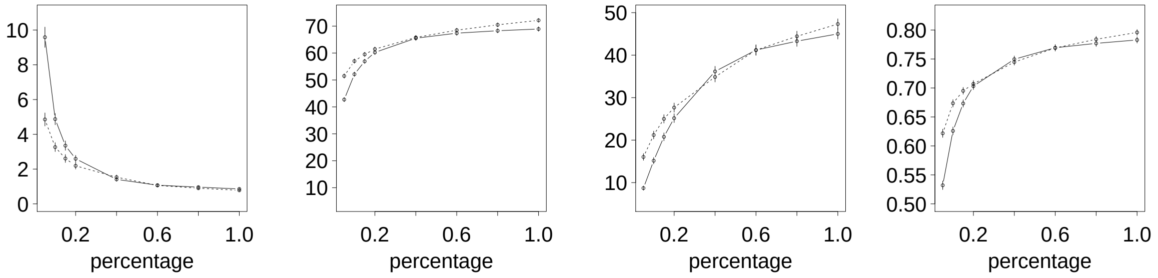

(e) False positive rate (f) Sensitivity (g) Positive predictive value (h) AUC-PR

(i)-(l) background order 2

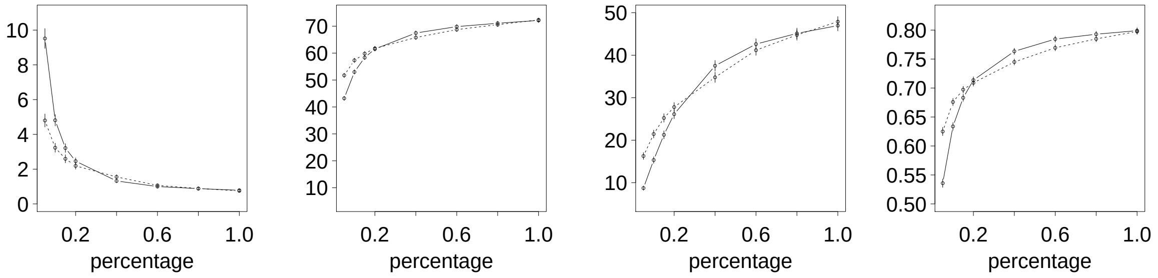

(i) False positive rate (j) Sensitivity (k) Positive predictive value (l) AUC-PR

(m)-(p) background order 3

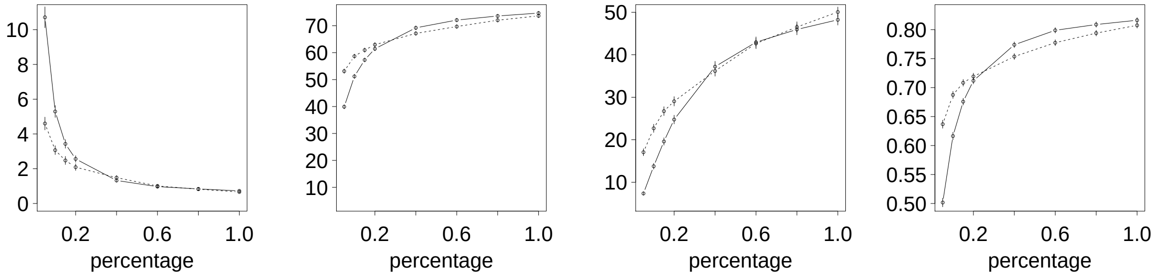

(m) False positive rate (n) Sensitivity (o) Positive predictive value (p) AUC-PR

Figure 2: Classification performance of generatively (solid line) and discriminatively (dashed line) trained Markov models on differently sized training data sets and different background orders for binding sites of the Sp1 transcription factor. We use a Markov model of order 1 in the foreground and Markov models of varying order in the background. We plot the four performance measures, false positive rate, sensitivity, positive predictive value, and area under the precision-recall curve (AUC-PR) against the percentage of the training data set used to estimate the parameters.
